# An improved method for identification of small non-coding RNAs in bacteria using support vector machine

**DOI:** 10.1038/srep46070

**Published:** 2017-04-06

**Authors:** Ranjan Kumar Barman, Anirban Mukhopadhyay, Santasabuj Das

**Affiliations:** 1 Biomedical Informatics Centre, National Institute Of Cholera and Enteric Diseases, Kolkata, West Bengal, India; 2 Department of Computer Science and Engineering, University of Kalyani, Kalyani, West Bengal, India; 3 Division of Clinical Medicine, National Institute of Cholera and Enteric Diseases, Kolkata, West Bengal, India

## Abstract

Bacterial small non-coding RNAs (sRNAs) are not translated into proteins, but act as functional RNAs. They are involved in diverse biological processes like virulence, stress response and quorum sensing. Several high-throughput techniques have enabled identification of sRNAs in bacteria, but experimental detection remains a challenge and grossly incomplete for most species. Thus, there is a need to develop computational tools to predict bacterial sRNAs. Here, we propose a computational method to identify sRNAs in bacteria using support vector machine (SVM) classifier. The primary sequence and secondary structure features of experimentally-validated sRNAs of *Salmonella* Typhimurium LT2 (SLT2) was used to build the optimal SVM model. We found that a tri-nucleotide composition feature of sRNAs achieved an accuracy of 88.35% for SLT2. We validated the SVM model also on the experimentally-detected sRNAs of *E. coli* and *Salmonella* Typhi. The proposed model had robustly attained an accuracy of 81.25% and 88.82% for *E. coli* K-12 and *S*. Typhi Ty2, respectively. We confirmed that this method significantly improved the identification of sRNAs in bacteria. Furthermore, we used a sliding window-based method and identified sRNAs from complete genomes of SLT2, *S*. Typhi Ty2 and *E. coli* K-12 with sensitivities of 89.09%, 83.33% and 67.39%, respectively.

Bacterial small non-coding RNAs (sRNAs) are transcripts that instead of encoding proteins, function directly at the level of RNA in the cells^1^,^2^. They are usually 50–250 nucleotides in length. sRNAs have appeared as key regulators of gene expression in pathogenic bacteria^3^. They were found to be involved in diverse biological processes, including bacterial virulence^4^,^5^, oxidative stress response^6^ and cell to cell communication in quorum sensing^7^. Recent advances in high throughput techniques, such as RNA sequencing (RNA-Seq)^8^,^9^ and tilling arrays^10^ have identified and characterized a number of sRNAs and provided valuable insights into bacterial physiology. However, experimental identification of sRNAs at a large scale is still lagging for several species. There is an urgent need to develop efficient computational tools for identifying sRNAs.

Several computational methods were developed in the recent past for the identification of sRNAs in bacteria using comparative genomics^11^,^12^,^13^,^14^, primary sequence^15^,^16^ and secondary structure^17^,^18^,^19^ features. QRNA^11^ used pairwise alignments to identify novel sRNAs in bacteria. This technique employed a pair hidden Markov models (pair-HMMs) and a pair stochastic context-free grammar (pair-SCFG) to find structured RNA (RNA), coding RNA (COD) or something else (OTH). RNA, COD and OTH models assumed that mutation pattern is significantly conserved in homologous RNA secondary structures, aligned sequences encode homologous proteins and mutations occur in simple position-independent manner, respectively. Alifoldz^12^ introduced combination of free energy and covariance to discriminate functional from random RNAs. MSARI^13^ algorithm applied the idea of detecting conserved RNA secondary structures among candidate orthologs by multiple sequence alignment. This program used RNAFOLD^20^ to predict the RNA secondary structure and CLUSTALW^21^ to make sequence alignment. zMFold^14^ offered a new shuffling program in perl (shuffle-pair.pl) for pairwise alignments that simultaneously preserves key features of the alignment. It used alignment dataset along with real and shuffled genomic sequences as inputs for a panel of published tools to identify novel non-coding RNA. Carter *et al*.^15^ developed a machine learning approach using neural network (NN) and support vector machine (SVM) to predict novel functional RNAs in the genomic sequence. The authors calculated twenty sequence compositions (mono (4) and di (16) nucleotide) and six structural motif features for all sequence windows and used them in training and testing of NN and SVM. Klein *et al*.^16^ proposed a screening technique for identifying novel non-coding RNAs by using both GC content bias and QRNA-based comparative analysis. RNAz2^17^ identified thermodynamically stable and evolutionary conserved RNA secondary structures in multiple sequence alignments and subsequently filtered candidate sRNAs. It calculated thermodynamic stability in terms of z-score and structure conservation index (SCI) to identify thermodynamically stable and evolutionary conserved RNA secondary structures, respectively. Dynalign II^18^, an update of Dynalign^19^, is a software package for prediction of the common secondary structure of two RNA homologs by predicting inserted domains into dynamic programming algorithm. However, a major shortcoming of the available computational methods to identify sRNAs is that most of them favour either sensitivity or specificity, but not both^22^ and generate either high number of false positives or false negatives. This underscores the necessity to develop a tool that will achieve higher accuracy to identify sRNAs with nearly equal sensitivity and specificity (where both false positive and false negative rates would be very low). Machine learning techniques have been extensively used to identify different classes of small non-coding RNA molecules, namely microRNAs (miRNAs)^23^,^24^,^25^,^26^,^27^,^28^,^29^,^30^,^31^,^32^,^33^,^34^ and transfer RNAs^23^,^24^. However, SVM^25^,^26^,^27^,^28^,^29^,^30^,^31^,^32^ rather than random forest (RF)^33^,^34^ and neural network (NN)^35^,^36^ were used more widely. Several excellent reviews are also available on this topic^37^,^38^,^39^,^40^.

In this study, we introduce an SVM classifier with 10-fold cross-validation technique^41^ that incorporates the primary sequence and secondary structure features of sRNAs to efficiently identify them in bacteria. Experimentally-validated sRNAs of *Salmonella* Typhimurium LT2 (SLT2) have been used to develop an optimum SVM model for identifying potential new sRNAs. The proposed SVM model efficiently identifies sRNAs with nearly equal sensitivity and specificity. We have also validated our proposed SVM model on other experimentally- validated sRNAs of *Escherichia coli (E. coli*) K-12 and *Salmonella* Typhi (*S*. Typhi) Ty2. In addition to this, we have applied sliding window-based method to identify sRNAs from the complete genome of a particular strain. All the source code, help file and proposed best SVM model is freely available at http://www.bicniced.org/RKB_profile.htm.

## Results

### Selection of best features

Different features and their combinations were used to achieve greater accuracy with nearly equal sensitivity and specificity. The secondary structure features were found to perform poorer as compared with the primary sequence features (Table 1). The tri-nucleotide composition (64) and all nucleotides composition (84, (4-mono + 16-di + 64-tri)) features performed slightly better than other primary sequence combination features. The tri-nucleotide and all nucleotides composition features achieved accuracies of 88.19% (sensitivity of 84.61% and specificity of 91.78%) and 87.92% (sensitivity of 89% and specificity of 86.83%) at threshold values of 0.3 and −0.2, respectively.

### Classification performance on imbalanced datasets

We tested the performance of our proposed method on imbalanced datasets also, to closely resemble real-world scenarios, where size of negative dataset is more than the positive dataset. As shown in Table 2, tri-nucleotide composition feature performed somewhat better when the negative dataset was double the size of the positive dataset. The tri-nucleotide composition feature (P:N = 1:2) achieved an accuracy of 88.35% with sensitivity of 85.11% and specificity of 91.58% at a threshold value of 0.0.

### Kernel-wise performance of SVM

We tested different kernels of SVM and combinations of parameters related to them along with other performance measures to achieve the best accuracy. As shown in Table S1, linear (accuracy of 85.53% with sensitivity of 84.50% and specificity of 86.56%), polynomial (accuracy of 87.79% with sensitivity of 84.56% and specificity of 91.03%) and RBF (accuracy of 88.35% with sensitivity of 85.11% and specificity of 91.58%) kernels of SVM performed better than sigmoid kernel (accuracy of 65.26% with sensitivity of 31.33% and specificity of 99.19%). The RBF kernel performed slightly better than the linear and polynomial kernels.

### Comparison of proposed SVM method with other machine learning methods

We compared our proposed SVM method with other frequently used machine learning methods like random forest and multilayer perceptron. As shown in Table 3, the proposed SVM method performed (accuracy of 88.35% with sensitivity of 85.11% and specificity of 91.58%) marginally better than random forest (accuracy of 81.18% sensitivity of 68.48% and specificity of 95.88%) and multilayer perceptron (accuracy of 85.71% sensitivity of 81.87% and specificity of 89.56%).

### Comparison with other basic computational method for identifying sRNAs

We compared the performance of our proposed model on SLT2 dataset with that of the other methods as estimated previously by Arnedo *et al*.^22^ with the same dataset. As shown in Table 4, our SVM model attained an accuracy of 88.35% with sensitivity of 85.11% and specificity of 91.58%. In contrast, all other methods except zMFold favoured specificity over sensitivity and performed poorly in terms of accuracy, sensitivity and specificity (Table 4). Arnedo *et al*.^22^ achieved an accuracy of 72.5% and sensitivity and specificity of 67% and 78%, respectively, which was inferior to our results. Thus, the data clearly indicate that our proposed method performed the best among different techniques to predict sRNA.

### Validation of proposed SVM model on others experimentally verified sRNAs

For the purpose of validation, we applied the SVM model to experimentally-verified sRNAs of other bacteria, which were not used in the original training and testing datasets. Our method achieved accuracies of 81.25% (sensitivity 73.75% and specificity 88.75%) and 88.82% (sensitivity 89.47% and specificity 88.16%) for sRNAs of *E. coli* K-12 and *S.* Typhi Ty2, respectively (Table 5). The result showed that the proposed model attained similar performance on SLT2, *E. coli* K-12 and *S.* Typhi Ty2 datasets.

### Performance on genome-wide identification of sRNAs

Finally, we applied sliding window-based approach to identify sRNAs from the complete genomes of *S.* Typhimurium LT2, *S.* Typhi Ty2 and *E. coli* K-12 using the SVM model we developed. As shown in Tables 6 and S2–S4, the sliding window-based method achieved comparable efficacy as the SVM model applied to known sRNA sequences with sensitivities of the former being 89.09%, 83.33% and 67.39% compared with the corresponding values for the latter being 85.11%, 89.47% and 73.75% for SLT2, *S.* Typhi Ty2 and *E. coli* K-12, respectively.

## Discussion

The role of sRNAs is more diverse than anticipated. Therefore, sRNA identification is of particular interest. The experimental techniques are ideal for the identification of sRNAs and exploration of their role in individual species. However, optimal techniques are lacking for many species. This limitation may be complemented by computational methods.

Several computational methods have been introduced over the years to identify sRNAs of bacteria, but most of them show poor performance in identifying positive and negative sRNAs simultaneously. Thus, methods such as MSARi, RNAz2, vsFold, Alifoldz and dynalign showed good performance in identifying negative sRNAs (non-sRNAs) identification, but poor ability in identifying positive sRNAs (Table 4). On the other hand, zMFold was efficient in identifying positive, but not negative sRNAs. As a result, these methods generated either a high number of false negatives (FN) or false positives (FP). Other tools like QRNA and the one proposed by Arnedo *et al*. showed slightly better performance for the identification of both positive and negative sRNAs simultaneously, but their accuracies were less than optimums.

In this study, we introduced a computational method to identify sRNAs of bacteria using SVM. The proposed SVM model simultaneously minimized the false negatives (FN) and false positives (FP). As a result, the model achieved decent accuracy with nearly equal sensitivity and specificity (Tables 1, 2, 3 and 4). This method showed significantly better performance as compared the existing techniques (Table 4). In addition, our method showed that simple nucleotide sequence features (tri-nucleotide) can efficiently predict sRNAs in bacteria (Tables 1 and 2). Finally, we applied the proposed SVM model to *E. coli* K-12 and *S.* Typhi Ty2 datasets for the purpose of validation. Our model achieved similar performance to SLT2 on *E. coli* K-12 and *S.* Typhi Ty2 datasets that were not used in the training and testing datasets.

To identify sRNAs form complete genome, we used sliding window-based approach. This method achieved good sensitivity for experimentally verified SLT2 and *S.* Typhi Ty2 datasets. However the performance for *E. coli* K-12 dataset was inferior to this with sensitivity of 67.39%. It was poorer compared with the SVM-based method we developed that achieved a sensitivity of 73.75% for *E. coli* K-12 dataset. Another reason for such difference might be attributed to the fact that 42.5% of experimentally verified sRNAs of *E. coli* K-12 are overlapping with the protein coding regions, whereas similar overlap for experimentally verified sRNAs of SLT2 and *S.* Typhi Ty2 was only 9.34% and 21.05%, respectively. We also found that the median of 80 experimentally-validated sRNAs of *E. coli* K-12 is 110 nucleotides. Therefore, we tried with the new window size of 110 and step size of 40 nucleotides for complete genome of *E. coli* K-12 dataset. The sliding window-based approach with the new parameters achieved the sensitivity of 71.74% for *E. coli* K-12 dataset, whereas window size of 145 and step size of 45 achieved a sensitivity of 67.39%. These results indicate that proper selection of window and step size may improve the performance of identification of sRNAs from the complete genome.

The primary goal of the present study was to predict small non-coding RNAs (sRNAs) from complete genome sequences of bacteria. Hence, we searched for simple sequence features, which could efficiently identify sRNAs. We found that simple tri-nucleotide composition feature can efficiently predict sRNAs. Literature search identified six sequence-derived features, such as spectrum profile, mismatch profile, subsequence profile, position-specific matrix, pseudo dinucleotide composition and local structure-sequence triplet elements, which can predict piwi-interacting RNAs (piRNA)^42^,^43^. We will use the above features in our future study to further improve the prediction model. We encountered several challenges to construct a webserver for predicting sRNAs from complete genomes. In the section on genome-wide identification of sRNAs using sliding windows technique of the manuscript, we had to collect protein coding table of a particular bacteria from the NCBI webserver. However, we faced difficulties in automatically downloading and formatting protein coding table from NCBI. In addition, all the steps to predict sRNAs from the complete genome would take few hours to complete. Hence, instead of creating a web server, we have deposited all the source codes at http://www.bicniced.org/RKB_profile.htm. We have also created user guide documents (HelpForPredictingsRNAsByBarmanetal.pdf and HelpForParsingCDS.pdf) to describe the steps to predict sRNAs from the complete genome. The user can predict sRNAs from the complete genome of bacteria of interest by using their local desktop.

## Methods

### Data collection

Experimentally validated 193 sRNAs of SLT2 were collected from Arnedo *et al*.^22^. These authors originally collected sRNAs of SLT2 from RFAM database^44^ as well as previously available literatures^45^,^46^,^47^,^48^,^49^. We retrieved a table with name, source of identification, start and end position of sRNAs from the published article (Supplementary Table S5)^22^. We had downloaded the complete genome sequence of SLT2 (http://www.ncbi.nlm.nih.gov/nuccore/16763390?report=fasta) and have extracted the exact sRNA sequences from it using the information about the start and end positions of particular sRNAs. We found that 11 out 193 sRNAs were redundant at the sequence levels except the start and the end positions. Since we planned to predict sRNAs using their primary sequences, we removed the redundant sRNAs from the SLT2 dataset. Thus, we finally used 182 experimentally-validated sRNAs of SLT2 as the positive dataset (Supplementary Table S6).

We used shuffleseq^50^ program to randomly shuffle the bases of complete genome sequence of SLT2 without affecting the composition. We generated ten negative (non-sRNAs) datasets by shuffling the complete genome sequence of SLT2 ten times and using the information on the start and end positions of the positive sRNAs (Supplementary Tables S7–S16). Furthermore, we plotted Venn-diagram using Venny webserver^51^ to ensure that all negative datasets were unique and different from the positive dataset as well as from each other (Supplementary Figures S1–S4).

In order to validate our proposed SVM model in other bacteria, we collected experimentally validated sRNAs of *E. coli* K-12 (80) and *S*. Typhi Ty2 (38) from Raghavan *et al*.^52^ and Perkins *et al*.^8^, respectively. The negative datasets of *E. coli* K-12 and *S*. Typhi Ty2 were generated using the same shuffleseq program as described above.

### 10-fold cross-validation

We used 10-fold cross-validation to estimate the performance of our proposed SVM model. In 10-fold cross-validation, the whole dataset was divided into 10 equal (nearly equal)-sized folds. Training and testing were repeated ten times so that each time a different fold was used for testing, while the remaining 9 folds were used for training. The overall performance of the proposed SVM model was calculated using average performance over 10 folds.

### Features

We used different nucleotide composition and secondary structure features for training and testing of SVM. A total of 84 nucleotides composition (4 for mono-nucleotide, 16 for dinucleotide and 64 for tri-nucleotide composition) and 3 secondary structure features (stem, loop and minimum free energy) were used as described below.

Mono-nucleotide composition: Mono-nucleotide composition of all 4 nucleotides was calculated using the following equation:


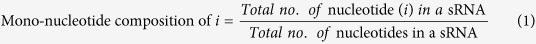


where *i* denotes any nucleotide “A” or “T” or “G” or “C”.

Di-nucleotide composition: Di-nucleotide composition of all 16 di-nucleotides was calculated using the following equation:


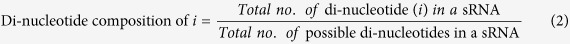


where *i* denotes any di-necleotide among 16 di-nucelotides (“AA”, “AT”, “AG”, “AC”, …. “CC”).

Tri-nucleotide composition: Tri-nucleotide composition of all 64 tri-nucleotides was calculated using the following equation:


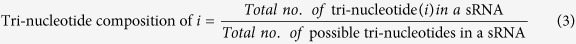


where *i* denotes any tri-necleotide among 64 tri-nucelotides (“AAA”, “ATA”, “AGA”, …, “CCC”).

Stem, loop and minimum free energy (MFE): RNAFOLD^20^ was used to calculate the number of stems, loops and minimum free energy (MFE) from the predicted secondary structure of individual sRNAs and non-sRNAs.

### SVM Classifier

Support Vector Machine (SVM) classifier was used to identify sRNAs and non-sRNAs. We employed SVM^light^ tool provided by T. Joachims^53^, which allows user to select different kernels and parameters to find a decision surface that maximizes the margin between data points of two classes (sRNAs and non-sRNAs). We have tested different kernels of SVM and the combinations of their respective parameters to optimize the performance. We have tested nearly 4000 combination of parameters (c [trade-off between training error and margin], j [Cost factor] for linear; d (d parameter in polynomial function), c, j for polynomial; g [gamma], c, j for RBF; and s [s parameter in sigmoid function], c, j, r [parameter c in sigmoid function] for sigmoid kernel) in each case and reported only the best one in the result section.

### Welch’s *t*-test

We employed Welch’s two sample *t*-test^54^ to find subset of nucleotide composition features that were significantly different between sRNAs and non-sRNAs (Welch’s two sample *t*-test p value < 0.05). We found that 13 nucleotide composition features in sRNAs were significantly different from non-sRNAs, while in case of non-sRNAs, 25 nucleotide composition features were found to be significantly different from sRNAs (Supplementary Table S17).

### Performance measures

Threshold-dependent performance measures of binary classification problem including sensitivity (Recall), specificity, accuracy, positive predictive value (PPV or precision), Mathew’s correlation coefficient (MCC) and F1 score were calculated using the following equations:


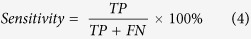



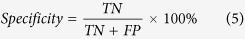



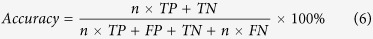



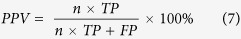



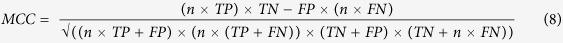


where *n* denotes the ratioof negative and positive datasets size


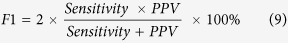


where, True Positive (TP): sRNAs are correctly identified as sRNAs.

False Positive (FP): non–sRNAs are incorrectly identified as sRNAs.

True Negative (TN): non–sRNAs are correctly identified as non–sRNAs.

False Negative (FN): sRNAs are incorrectly identified as non–sRNAs.

Threshold-independent performance measure like area under receiver operating characteristic curve (ROC) plot (AUC) was computed for all cases.

### Genome-wide identification of sRNAs using sliding windows technique

We found that most of the experimentally-verified sRNA (~85%) sequences fall in the inter-genic regions of the genome. In order to derive inter-genic region of a particular genome, we collected the complete genome sequence and the protein-coding table of the corresponding strain from the NCBI genome database. If the protein-coding table was not directly available for a certain strain, we parsed the coding table from NCBI. All the coding regions were excluded and only the inter-genic regions were retained for further studies. Additionally, the inter-genic regions of lengths less than 50 nucleotides were also excluded.

We exploited sliding window-based approach to identify sRNAs from the inter-genic regions. The selection of the window and step size plays a crucial role in identifying sRNAs from the complete genome. Previously, window size of 100 to 200 and step size of 40 to 50 nucleotides were used to predict sRNAs in prokaryotes^55^,^56^,^57^. We selected a window size of 145 and step size of 45 nucleotides, since the median lengths of experimentally-verified sRNAs of SLT2 (182 sRNAs) and Bacterial Small Regulatory RNA Database (BSRD) (897 sRNAs) are 145.5 and 145, respectively. We generated every possible window for all the inter-genic regions of a bacterial genome and calculated the tri-nucleotide composition features of the windows. We then predicted sRNAs for each possible window by using our proposed SVM model. Finally, average prediction score of the windows corresponding to an experimentally-verified sRNA was calculated. If the average prediction score was greater than 0 (threshold value), we treated it as positive or truly predicted one.

## Additional Information

**How to cite this article:** Barman, R. K. *et al*. An improved method for identification of small non-coding RNAs in bacteria using support vector machine. *Sci. Rep.* **7**, 46070; doi: 10.1038/srep46070 (2017).

**Publisher's note:** Springer Nature remains neutral with regard to jurisdictional claims in published maps and institutional affiliations.

## Supplementary Material

Supplementary Tables

Supplementary Figures

## Figures and Tables

**Table 1:** Performance measures on different combination of features in SLT2 dataset, using RBF kernel of SVM.

Features set	Vector length	P(+): N(−)	Threshold	Sensitivity (%)	Specificity (%)	Accuracy (%)	PPV (%)	MCC	F1 score (%)	AUC
**Primary sequence features**										
All nucleotides composition	**84**	**1**:**1**	**−0.20**	**89.00**	**86.83**	**87.92**	**87.75**	**0.76**	**88.37**	**0.929**
Tri-nucleotide composition	**64**	**1**:**1**	**0.30**	**84.61**	**91.78**	**88.19**	**91.75**	**0.77**	**88.04**	**0.938**
Mono and di-nucleotide composition	20	1:1	0.00	76.94	85.78	81.36	85.42	0.64	80.96	0.888
Di-nucleotide composition	16	1:1	0.00	78.06	86.83	82.44	87.02	0.66	82.29	0.884
Mono-nucleotide composition	4	1:1	0.10	78.50	64.39	71.44	68.82	0.44	73.34	0.754
Best features out of all nucleotides composition features (84), using Welch Two Sample t-test P value < 0.05.	38	1:1	0.00	88.89	82.50	85.69	83.95	0.72	86.35	0.906
Best features out of all nucleotides composition features (84), using Welch Two Sample t-test P value < 0.05. Here, nucleotides composition were significantly higher in positive (+ve) set rather negative (−ve) set.	13	1:1	0.20	85.06	78.72	81.89	80.75	0.65	82.84	0.893
Best features out of all nucleotides composition features (84), using Welch Two Sample t-test P value < 0.05. Here, nucleotides composition were significantly higher in negative (−ve) set rather positive (+ve) set.	25	1:1	0.10	82.61	87.28	84.94	87.27	0.70	84.88	0.890
**Secondary structure features**										
Stem, Loop and Minimum free energy (MFE)	3	1:1	0.00	61.17	80.83	71.00	77.08	0.43	68.21	0.732
Stem and Loop	2	1:1	−0.30	43.94	69.89	56.92	59.14	0.15	50.42	0.587
Stem	1	1:1	0.70	74.06	34.56	54.31	53.02	0.12	61.80	0.535
Loop	1	1:1	0.30	92.94	43.61	68.28	62.64	0.43	74.84	0.667
MFE	1	1:1	−0.10	76.39	36.72	56.56	54.94	0.14	63.92	0.547

Optimal parameter sets were used for respective combination of features.

**Table 2:** SVM performance measures on balance and imbalanced SLT2 datasets.

Best feature sets	Vector length	P(+): N(−)	Threshold	Sensitivity (%)	Specificity (%)	Accuracy (%)	PPV (%)	MCC	F1 score (%)	AUC
All nucleotides composition features	84	1:1	−0.20	89.00	86.83	87.92	87.75	0.76	88.37	0.929
Tri-nucleotide composition features	64	1:1	0.30	84.61	91.78	88.19	91.75	0.77	88.04	0.938
All nucleotides composition features	84	1:2	0.00	84.00	89.64	86.82	89.21	0.74	86.53	0.924
Tri-nucleotide composition features	**64**	**1**:**2**	**0.00**	**85.11**	**91.58**	**88.35**	**91.21**	**0.77**	**88.06**	**0.937**
All nucleotides composition features	84	1:3	−0.10	82.89	90.30	86.59	89.79	0.74	86.20	0.931
Tri-nucleotide composition features	64	1:3	0.00	81.83	92.70	87.27	91.94	0.75	86.59	0.944
All nucleotides composition features	84	1:4	−0.10	81.89	91.28	86.58	90.51	0.74	85.98	0.921
Tri-nucleotide composition features	64	1:4	0.00	81.83	92.92	87.38	92.24	0.76	86.73	0.944
All nucleotides composition features	84	1:5	−0.30	81.89	91.14	86.52	90.32	0.74	85.90	0.928
Tri-nucleotide composition features	64	1:5	−0.50	88.44	86.50	87.47	86.83	0.75	87.63	0.943
All nucleotides composition features	84	1:10	−0.90	89.06	84.01	86.53	84.96	0.73	86.96	0.935
Tri-nucleotide composition features	64	1:10	−0.50	86.22	89.56	87.89	89.27	0.76	87.72	0.946

Optimal parameter sets were used for respective balance and imbalanced SLT2 datasets.

**Table 3:** Performance comparison of different machine learning methods.

Machine learning method	Best feature sets	Vector length	P(+): N(−)	Sensitivity (%)	Specificity (%)	Accuracy (%)	PPV (%)	MCC	F1 score (%)	AUC
SVM	Tri-nucleotide composition	**64**	**1:2**	**85.11**	**91.58**	**88.35**	**91.21**	**0.77**	**88.06**	**0.937**
Multilayer perceptron	Tri-nucleotide composition	64	1:2	81.87	89.56	85.71	88.69	0.71	85.14	0.908
Random forest	Tri-nucleotide composition	64	1:2	66.48	95.88	81.18	94.16	0.68	77.94	0.927

Optimal parameter sets were used for respective methods.

**Table 4:** Performance measures of the individual methods on SLT2 dataset.

Method	SLT2 Sensitivity (%)	SLT2 Specificity (%)	SLT2 Accuracy (%)
QRNA	59.00	71.00	65.00
Alifoldz	42.00	87.00	64.50
MSARi	2.00	100.00	51.00
zMFold	90.00	49.00	69.50
RNAz2	27.00	98.00	62.50
dynalign	28.00	86.00	57.00
vsFold	25.00	88.00	56.50
Arnedo *et al*.	67.00	78.00	72.50
**Proposed**	**85.11**	**91.58**	**88.35**

**Table 5:** Performance measures of proposed SVM model on experimentally verified sRNAs of others bacteria that are not used in training and testing datasets.

Bacterial strain	No. of sRNAs	P(+): N(−)	Sensitivity (%)	Specificity (%)	Accuracy (%)	PPV(%)	MCC	F1 score(%)	AUC
*E. coli* K-12	80	1:2	73.75	88.75	81.25	86.76	0.63	79.73	0.901
*S*. Typhi Ty2	38	1:2	89.47	88.16	88.82	88.31	0.78	88.89	0.926

**Table 6:** Performance of sliding windows based approach for identifying sRNAs from complete genome.

Dataset	No. of experimentally verified sRNAs	No. of sRNAs in intergenic regions	Positively predicted by proposed SVM model	% ofprediction
*Salmonella* Typhimurium LT2	182	165	147	89.09
*Salmonella* Typhi ty2	38	30	25	83.33
*E. Coli* K-12	80	46	31	67.39
