# Supplementary material for: An improved method for identification of small non-coding RNAs in bacteria using support vector machine
Source: Sci Rep. 2017 Apr 6;7:46070. doi: 10.1038/srep46070 (PMC5382675; doi:10.1038/srep46070)
Supplement: Supplementary Figures [file srep46070-s2.pdf]

An improved method for identification of small non-coding RNAs in bacteria using support vector machine

Ranjan Kumar Barman[1], Anirban Mukhopadhyay[3], Santasabuj Das[1,2]*

Supplementary Figures

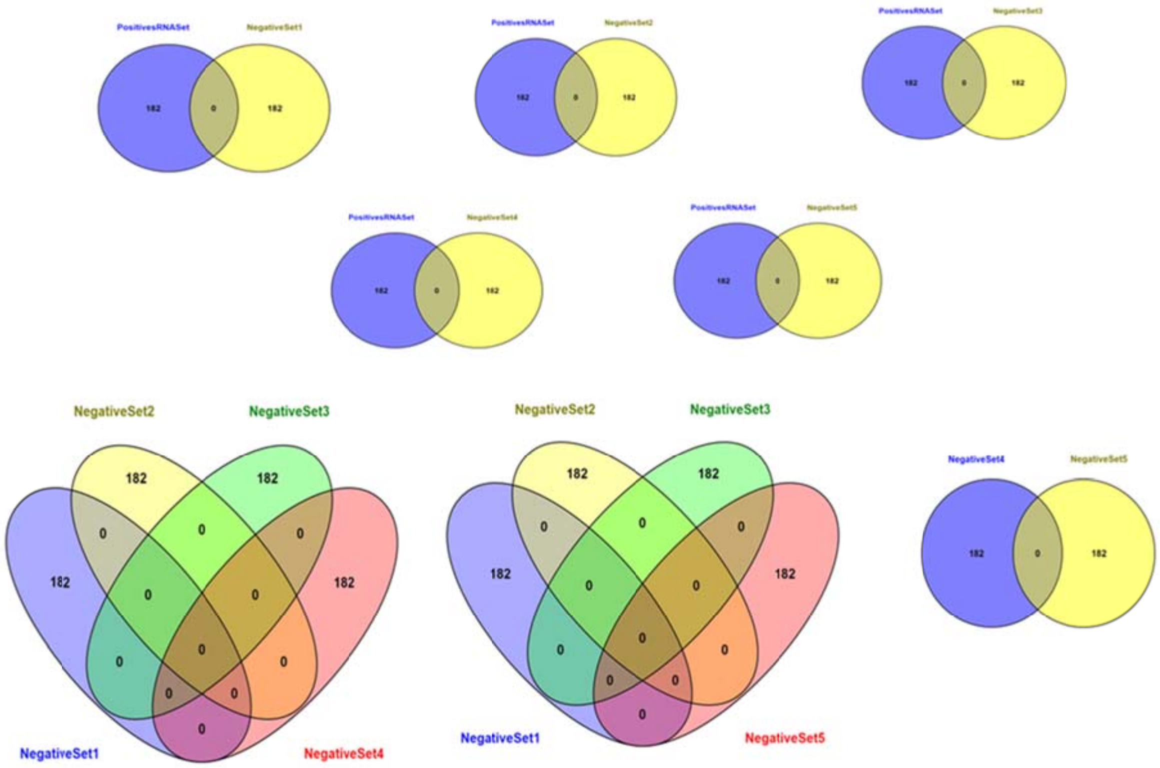

Figure S1: Venn-diagram of positive set and negative set 1, 2, 3, 4 and 5.

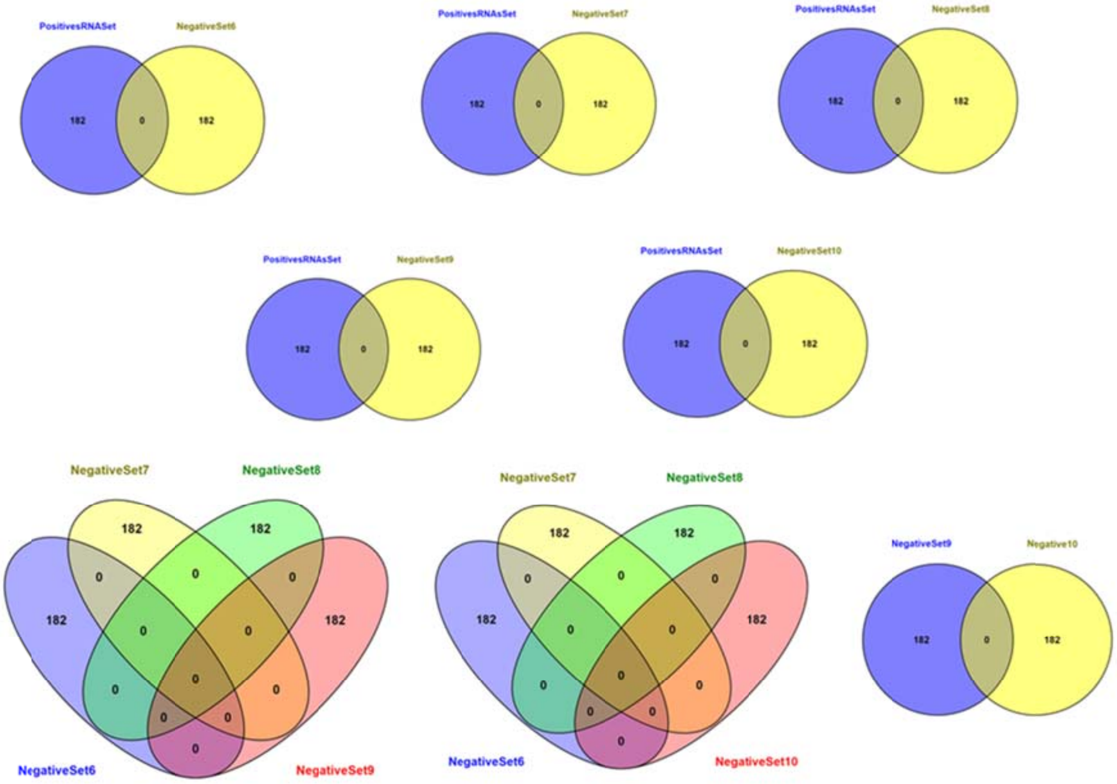

Figure S2: Venn-diagram of positive set and negative set 6, 7, 8, 9 and 10**.**

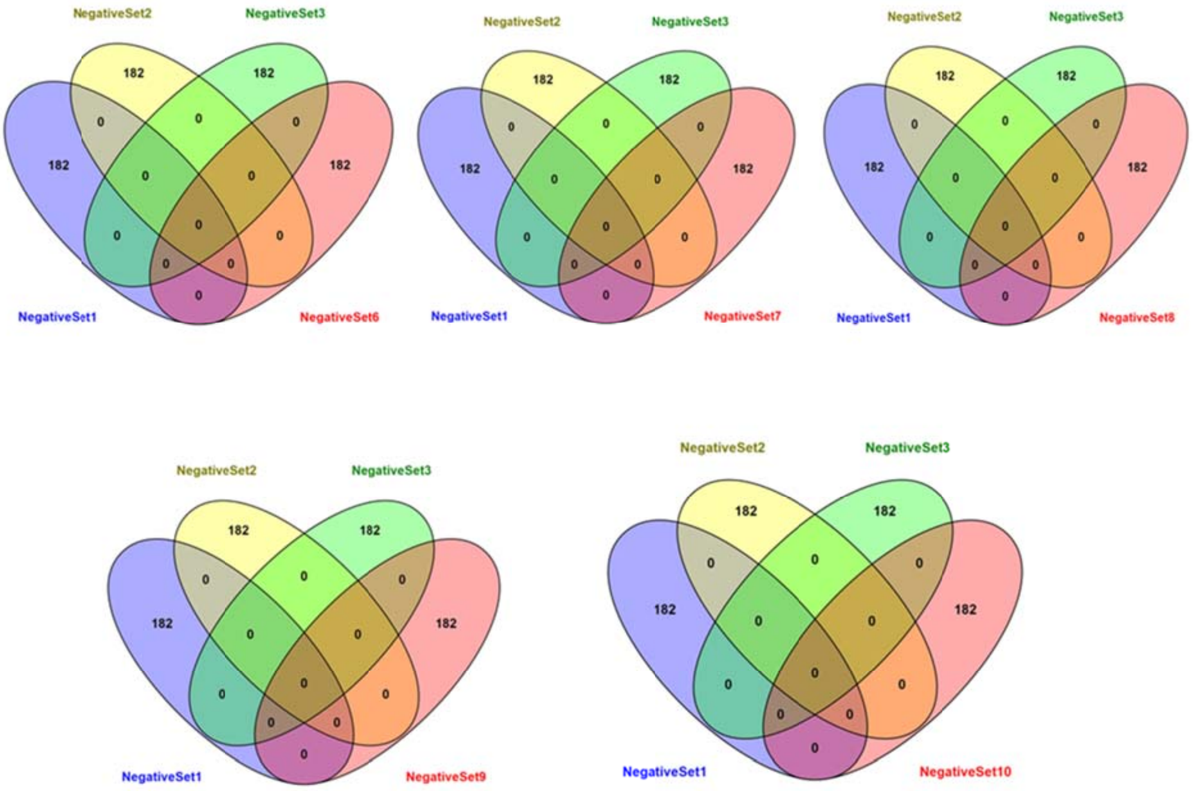

Figure S3: Venn-diagram of negative set 1, 2, 3 and negative set 6, 7, 8, 9, 10.

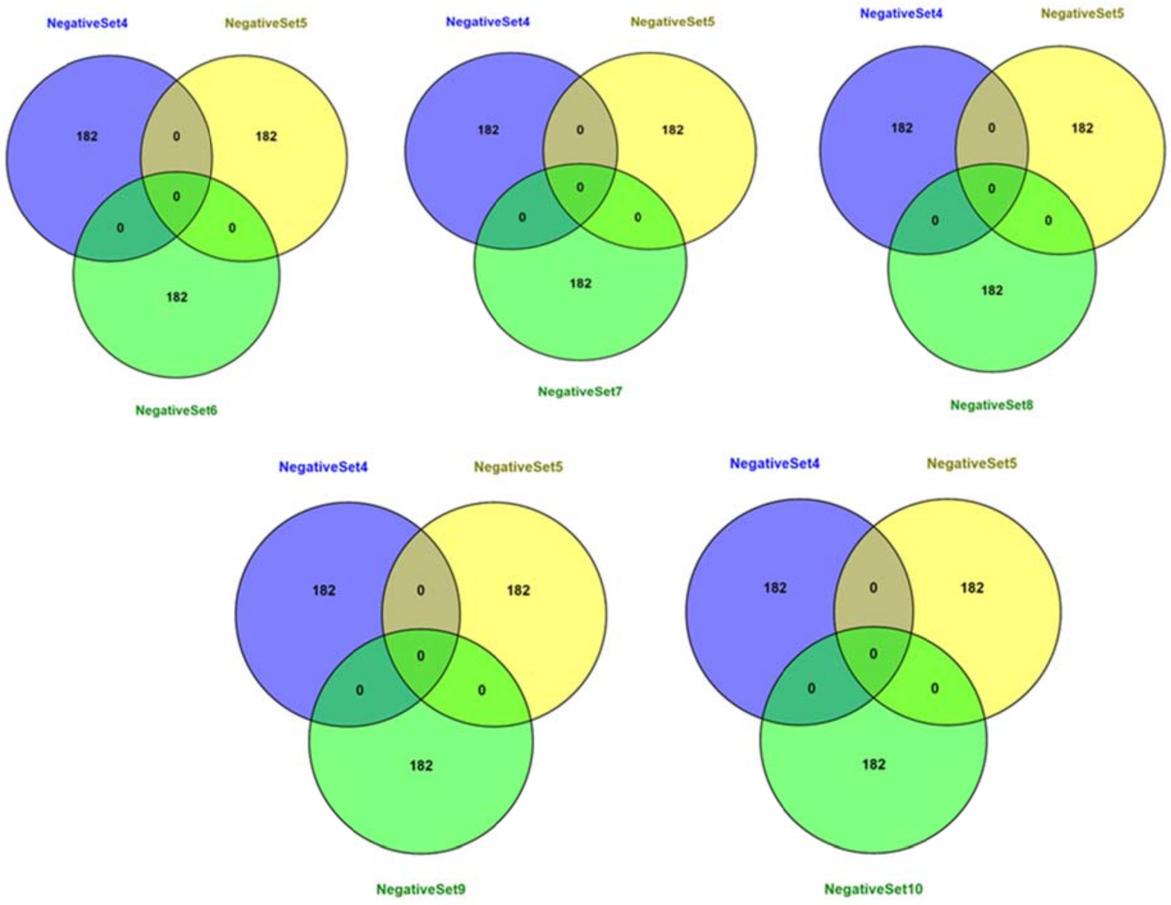

Figure S4: Venn-diagram of negative set 4, 5 and negative set 6, 7, 8, 9, 10.
